# Blood Viscoelasticity Measurement Using Interface Variations in Coflowing Streams under Pulsatile Blood Flows

**DOI:** 10.3390/mi11030245

**Published:** 2020-02-26

**Authors:** Yang Jun Kang

**Affiliations:** Department of Mechanical Engineering, Chosun University, 309 Pilmun-daero, Dong-gu, Gwangju 61452, Korea; jkang2011@chosun.ac.kr; Tel.: +82-62-230-7052

**Keywords:** viscoelasticity, microfluidic device, coflowing streams, interface, linear differential equation, two approximate factors

## Abstract

Blood flows in microcirculation are determined by the mechanical properties of blood samples, which have been used to screen the status or progress of diseases. To achieve this, it is necessary to measure the viscoelasticity of blood samples under a pulsatile blood condition. In this study, viscoelasticity measurement is demonstrated by quantifying interface variations in coflowing streams. To demonstrate the present method, a T-shaped microfluidic device is designed to have two inlets (a, b), one outlet (a), two guiding channels (blood sample channel, reference fluid channel), and one coflowing channel. Two syringe pumps are employed to infuse a blood sample at a sinusoidal flow rate. The reference fluid is supplied at a constant flow rate. Using a discrete fluidic circuit model, a first-order linear differential equation for the interface is derived by including two approximate factors (*F_1_* = 1.094, *F_2_* = 1.1087). The viscosity and compliance are derived analytically as viscoelasticity. The experimental results showed that compliance is influenced substantially by the period. The hematocrit and diluent contributed to the varying viscosity and compliance. The viscoelasticity varied substantially for red blood cells fixed with higher concentrations of glutaraldehyde solution. The experimental results showed that the present method has the ability to monitor the viscoelasticity of blood samples under a sinusoidal flow-rate pattern.

## 1. Introduction

Cardiovascular diseases (CVDs) occur without any symptoms and can lead to unexpected death [1]. In other words, blood clotting or abnormal blood flow contributes to vasculature blockages. Currently, biochemical properties (i.e., biomarkers [2,3] or DNA [4]) are used to diagnose CVDs. However, the biochemical approach has not been considered an effective tool for early detection of CVDs, because it does not provide information on blood flows or blood clotting. Instead of the biochemical approach, a biophysical approach is required to quantify abnormal blood flows in narrow-sized vessels. Blood samples collected from patients with CVDs or disorders exhibit changes in cells (i.e., red blood cells (RBCs) [5] or platelets [6]) or plasma proteins [3,7]. To detect CVDs effectively, it is necessary to quantify the contributions of cells or plasma proteins. Blood flows in microcirculation are determined by the mechanical properties of blood samples. In addition, blood vessel walls (i.e., shape and size) contribute to varying blood flows substantially. These properties include viscosity, elasticity, RBC aggregation, and RBC deformability. According to recent reports, a blood sample collected from a patient with CVD showed significantly different biophysical properties when compared with a normal blood sample [2,8,9]. For this reason, the mechanical properties of blood samples have been used to monitor the status or progress of CVDs. Additionally, the mechanical properties have been quantified under dynamic blood flows [10]. Under ex vivo closed-circuit conditions, blood flows vary periodically over time. When a flow regulator is integrated into the fluidic circuit, a periodic flow pattern is regulated to a constant flow pattern [11]. The viscosity of the blood sample is then quantified by using reverse flow-switching phenomena under a constant blood flow. Additionally, blood viscosity is obtained by monitoring the interface in coflowing streams, while the flow rates of the blood sample and reference fluid remain constant at the same flow rate with two syringe pumps [12,13]. However, because the blood sample includes viscoelasticity (viscosity and elasticity) under a periodic flow condition, it is necessary to quantify the viscoelasticity of the blood sample without a flow regulator (i.e., periodic flow rate).

Recently, a microfluidic platform has been suggested for effectively manipulating a small volume of blood sample in microfluidic channels. A microfluidic channel has been used to investigate hemorheological properties of blood samples. Under a microfluidic platform, several techniques have been suggested to quantify the viscoelasticity of blood samples. Guido et al. have measured membrane viscoelasticity by measuring the velocity and shape of a single RBC in converging or diverging flow [14]. Kim et al. measured RBC stretching with viscoelastic cell focusing [15]. The method was then used to characterize differences in RBC deformability. Lee et al. measured the viscosity and elasticity of blood samples by infusing steady and transient blood flows sequentially [16]. The viscosity and time constant were obtained sequentially by controlling the flow rate of the reference fluid and blood sample, respectively. Here, the time constant was quantified by monitoring temporal variation of a bridge channel filled with a human blood sample at a transient blood flow. The elasticity of the blood sample was quantified by using the linear Maxwell model (elasticity = viscosity/time constant). Kang reported that viscosity and elasticity of blood samples can be obtained sequentially under a periodic on–off blood flow condition [17]. Monitoring the interface in a coflowing stream enabled blood viscoelasticity to be obtained at an interval of a specific period. 

More recently, the author suggested a viscoelasticity measurement method under a sinusoidal blood flow rate (*Q_B_* (*t*)) (*Q_B_* (*t*) = *Q_α_* + *Q_β_*·sin (ω*t*), where *Q_α_* is the mean flow rate, *Q_β_* is the alternating flow rate, and *ω* is the angular frequency of syringe pump) [18]. While the blood sample is infused periodically, the viscoelasticity of the blood sample is obtained by monitoring the interface in coflowing streams and by calculating the pulsatility index (*PI*) (*PI* = 0.5 × (*Q_max_* - *Q_min_*)/*Q_ave_*, where *Q_max_* is the maximum flow rate, *Q_min_* is the minimum flow rate, and *Q_ave_* is the average flow rate). A first-order differential equation for coflowing streams is derived by constructing a simple discrete fluidic circuit. Here, using a conventional microelectromechanical system technique, a microfluidic device has rectangular shape with an extremely low aspect ratio (aspect ratio = depth/width = 4/250) which is devised to compensate for the boundary condition difference between a real physical model and a mathematical condition. First, the viscosity of the blood sample is calculated by averaging the equation over a single period. Second, instead of an analytical solution of the equation, the time constant is obtained from the expression of *PI*. Variations of velocity and interface are required simultaneously to find out time constant of interface. The elasticity is then obtained with a linear Maxwell model. However, the differential equation does not include a correction factor (*CF*), which makes it necessary to compensate for the boundary condition difference between the real model and mathematical model under coflowing streams [19]. Additionally, the previous study did not use the analytical expression to obtain the viscoelasticity of a blood sample. At last, the method required variations of blood velocity over time. Nonetheless, the previous method shows promise for quantifying the viscoelasticity of blood samples under a periodic blood flow-rate pattern. It is extremely difficult to obtain the viscoelasticity of a blood sample circulated under an ex vivo or in vivo condition (i.e., a real and complex situation). As a preliminary study, it is necessary to develop a simple method for measuring viscoelasticity under a single sinusoidal flow patterns with a syringe pump. 

In this study, to resolve these issues, a *CF* is inserted while deriving a differential equation for coflowing streams. Because the differential equation includes nonlinear terms, it is difficult to find an analytical solution. Conducting computational fluid dynamics (CFD) simulation enables an approximate expression of the *CF* to be obtained. Then, two approximation factors (*F_1_*, *F_2_*) are suggested and calculated to convert nonlinear terms into linear terms. Analytical expressions of the viscoelasticity of blood samples are obtained by solving the differential equation. Here, viscosity and compliance are derived analytically. To demonstrate the present method, a T-shaped microfluidic channel is used. When measuring velocity fields of blood sample, a T-shaped microfluidic channel is not required to align a microscopic image in the horizontal or vertical direction. It consists two inlets, one outlet, two guiding channels (blood sample channel, reference fluid channel), and one coflowing channel. Using two syringe pumps, a blood sample is infused into the blood sample channel with a sinusoidal flow-rate pattern. A reference fluid is supplied into the reference fluid channel at a constant flow rate. By monitoring the interface of both fluids in the coflowing channel, the viscosity and compliance are obtained at an interval of a specific period. As a performance demonstration, the present method was used to evaluate the contributions of period (*T*), diluents (plasma, 1x phosphate-buffered saline (PBS), and hematocrit (*Hct*) to viscoelasticity. The present method was then employed to quantify the viscoelasticity of a fixed blood sample prepared by adding fixed RBCs into plasma. 

## 2. Materials and Methods 

### 2.1. Blood Sample Preparation

According to the ethics committee of Chosun University Hospital (CHOSUN 2018-05-11), all experiments were conducted after ensuring that the experimental protocols were appropriate and humane.

Human concentrated RBCs and fresh frozen plasma (FFP) were purchased from the Gwangju–Chonnam blood bank (Gwangju, Korea) and were stored at 4 °C and −20 °C, respectively. Because the RBCs were preserved in citrate phosphate dextrose adenine (CPDA) as an anticoagulant solution, it was necessary to remove CPDA from the concentrated RBCs. The concentrated RBCs (~7 mL) were added into 1x PBS (pH 7.4, Gibco, Life Technologies, Carlsbad, CA, USA) (~7 mL) in a 15-mL tube. After the tube was inserted into a centrifugal separator (Allegra X-30R benchtop, Beckman Coulter, Brea, CA, USA), it was set to 4000 rpm and operated for 10 min. The diluted blood was separated into two layers: an upper layer (plasma), and a lower layer (RBCs). Pure RBCs were collected after removing liquid in the upper layer. Additionally, FFP was thawed at room temperature (25 °C). Plasma was filtered to remove cellular debris and unwanted white blood cells with a syringe filter (mesh size = 5 µm, Minisart, Sartorius, Göttingen, Germany). The RBCs and plasma were stored at 4 °C in a refrigerator before the blood test [20]. 

First, to evaluate the effect of the contribution of *Hct* and diluents (1x PBS, plasma) on the viscoelasticity of blood samples, blood samples with *Hct* = 30%, 40%, 50%, and 60% were prepared by adding normal RBCs into 1x PBS or plasma. Except in the experiment for evaluating the contribution of *Hct*, all blood samples were adjusted to *Hct* = 50%. Second, to fix normal RBCs chemically, three different concentrations of glutaraldehyde (GA) solution (*C_GA_* = 4, 8, and 12 µL/mL) were diluted by mixing GA solution (Grade II, 25% in H_2_0, Sigma-Aldrich, St. Louis, MO, USA) into 1x PBS. Normal RBCs were fixed for consistent measurement because RBCs needed to be unchanged over experimental time. To fix normal RBCs, normal RBCs were mixed with each concentration of GA solution for 10 min prior to washing them. Fixed RBCs were collected after a washing procedure. The fixed blood sample (*Hct* = 50%) was then prepared by adding the fixed RBCs into plasma. Here, to evaluate the contribution of fixed RBCs to viscoelasticity effectively, it was necessary to remain constant at a level of hematocrit (i.e., *Hct* = 50%).

### 2.2. Fabrication on a Microfluidic Device and Experimental Procedure

**Figure 1:**
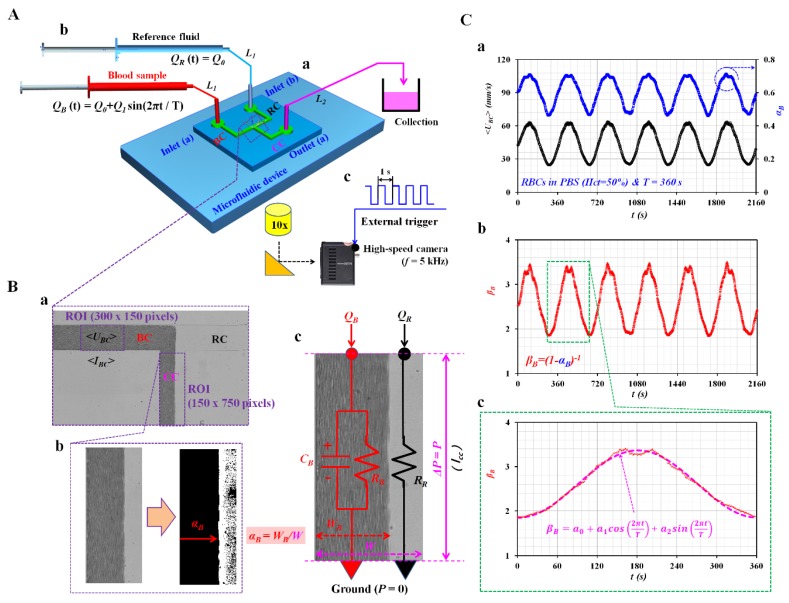
Proposed method for measuring blood viscoelasticity by monitoring the interface of both fluids in coflowing streams under a pulsatile blood flow rate. (**A**) Schematic diagram of the proposed method, including a microfluidic device, two syringe pumps, and an image acquisition system. (**a**) Microfluidic device composed of two inlets (a, b), one outlet (a), two guiding channels (blood sample channel (BC) and reference-fluid channel (RC)), and a coflowing channel (CC). (**b**) Two syringe pumps employed to supply blood sample and reference fluid into the corresponding inlets. (**c**) High-speed camera with a frame rate of 5 kHz employed to capture microscopic images at an interval of 1 s. (**B**) Quantification of interface in coflowing channel and its mathematical model with a discrete fluidic circuit. (**a**) Region of interest (ROI, 150 × 750 pixels) selected in coflowing channel for evaluating interface (*α_B_*) and ROI (300 × 150 pixels) selected in blood sample channel for evaluating averaged blood velocity (*<U_BC_>*) or averaged image intensity (*<I_BC_>*). (**b**) Image conversion from gray-scale image to binary-scale image by using digital image processing. Interface (*α_B_*) was obtained as *α_B_* = *W_B_*/*W*. *W_B_* and *W* represent blood-filled width and channel width, respectively. (**c**) Discrete fluidic circuit for mathematical representation of two fluids flowing in coflowing channel. Ground represented zero value of pressure (*P* = 0). (**C**) As a preliminary demonstration, a blood sample (*Hct* = 50%, RBCs suspended in 1x PBS) was supplied into inlet (a) at a sinusoidal flow rate (*Q_B_* (*t*) = 1 + 0.5 sin (2πt/360) mL/h); 1x PBS was supplied into inlet (b) at a constant flow rate of *Q_R_* (*t*) = 1 mL/h. (**a**) Temporal variations of *<U_BC_>* and *α_B_* with an elapse of time. (**b**) Temporal variations of *β_B_* = (1 − *α_B_*)^−1^ over time. (**c**) Extractions of three constants (*a_0_*, *a_1_*, and *a_2_*) of *β_B_* (*t*) = *a_0_* + *a_1_*·cos (ω·*t*) + *a_2_*·sin (ω·*t*) by conducting a curve-fitting technique for a single period of 360 s.

A T-shaped microfluidic device for measuring blood viscoelasticity consisted of two inlets (a, b), one outlet (a), two guiding channels (blood sample channel (BC), reference fluid channel (RC)), and one coflowing channel (CC), as shown in Figure 1A-a and Figure S1 (Supplementary Materials). The blood sample channel (width = 250 μm, length = 7500 μm) and reference fluid channel (width = 250 μm, length = 7500 μm) were connected to the coflowing channel (width = 250 μm, length = 9200 μm). Here, dimensions of a microfluidic channel were selected to measure velocity fields and blood viscosity accurately. First, velocity fields of blood flows were obtained accurately with microscopic images captured with at least 10× objective lens. Based on fields of view, channel width and length were selected suitably. Second, a rectangular channel with low aspect to ratio was preferred to measure blood viscosity effectively under coflowing method. The channel depth of the microfluidic device was fixed at 20 μm. 

Conventional microelectromechanical-system fabrication techniques (photolithography and deep reactive ion etching) were used to fabricate a master mold on a 4-inch silicon wafer. polydimethylsiloxane (PDMS) (Sylgard 184, Dow Corning, Midland, MI, USA) prepolymer and a curing agent were mixed at a ratio of 10:1. After the mold was fixed in a Petri dish, the PDMS mixture was poured on the master mold. Air bubbles in the PDMS were removed with a vacuum pump for 1 h. After curing the PDMS mixture in a convective oven (70 °C for 1 h), a PDMS block was peeled from the master mold. It was cut with a razor blade. Three ports (two inlets and one outlet) were punched with a biopsy punch (outer diameter = 1.2 mm). After oxygen–plasma treatment on the PDMS block and a glass slide with an oxygen–plasma system (CUTE-MPR, Femto Science Co., Gyeonggi, Korea), a microfluidic device was finally prepared by bonding the PDMS block on the glass slide.

As shown in Figure 1A-b, two polyethylene tubes (*L_1_*, inner diameter = 500 μm, thickness = 500 μm, and length = 300 mm) were connected from two disposable syringes (~1 mL) to two inlets (a and b). The other polyethylene tube (*L_2_*, inner diameter = 500 μm, thickness = 500 μm, and length = 200 mm) was connected from an outlet (a) to a waste collection unit. To remove air bubbles in the channels and avoid nonspecific binding of plasma proteins to the inner surfaces of the channels, all channels were filled completely with bovine serum albumin (BSA) solution of *C_BSA_* = 2 mg/mL through outlet (a) with a disposable syringe. After 10 min, all channels were rinsed then filled with 1x PBS. After two disposable syringes (~1 mL) were filled with blood sample (~1 mL) and 1x PBS (~1 mL), they were installed into two syringe pumps (neMESYS, Cetoni GmbH, Germany). The blood sample was supplied into inlet (a) at a sinusoidal flow rate (*Q_B_* (*t*) = *Q_0_* + *Q_1_*·sin (2πt/*T*)). *Q_0_* and *Q_1_* are the average and amplitude of the sinusoidal flow rate. Additionally, *T* represents the period. Reference fluid (1x PBS) was supplied into inlet (b) at a constant flow rate (*Q_R_* (*t*) = *Q_0_*).

As shown in Figure 1A-c, the microfluidic device was positioned on an optical microscope (BX51, Olympus, Tokyo, Japan) equipped with a 10× objective lens (NA = 0.25). A high-speed camera (FASTCAM MINI, Photron, USA) was used to capture microscopic images of the blood sample and 1x PBS flowing in microfluidic channels. It offered a spatial resolution of 1280 × 1000 pixels. Each pixel corresponded to 10 µm. With a function generator (WF1944B, NF Corporation, Yokohama, Japan), a pulse signal with a period of 1 s triggered the high-speed camera. Microscopic images were sequentially captured at a frame rate of 5 kHz. All experiments and blood sample preparations were conducted at a room temperature of 25 °C.

### 2.3. Quantification of Interface (α_B_), Averaged Blood Velocity (<U_BC_>), and Averaged Image Intensity (<I_BC_>)

Variations of the interface in the coflowing channel were used to quantify the viscoelasticity of the blood sample. Additionally, the image intensity and velocity fields of the blood sample flowing in the blood sample channel were obtained to evaluate the erythrocyte sedimentation rate (ESR) that occurred in the driving syringe while the blood flow rate was controlled by the syringe pump. First, to obtain the interface between the blood sample and 1x PBS in the coflowing channel, a specific ROI of 150 × 750 pixels was selected in the coflowing channel, as shown in Figure 1B-a. To obtain the interface in the coflowing channel effectively, a gray-scale image was converted into a binary-scale image by adopting Otsu’s method [21]. As shown in Figure 1B-b, the blood-filled width (*W_B_*) over the ROI was calculated by using a commercial software package (MATLAB 2019, MathWorks, Natick, MA, USA). The interface (*α_B_*) was obtained as *α_B_* = *W_B_*/*W*. Here, *W* is the channel width of the coflowing channel. Second, to monitor variations of blood flow rate supplied from the syringe pump, velocity fields of the blood sample flowing in the blood sample channel were obtained by conducting a time-resolved micro particle image velocimetry (micro-PIV) technique. A specific ROI of 300 × 150 pixels was selected in the blood sample channel. The size of the interrogation window was 32 × 32 pixels. The window overlap was 50%. The obtained velocity fields were validated with a median filter. The averaged velocity (<*U_BC_*>) was calculated as an arithmetic average of *U_BC_* distributed over the ROI. Third, to evaluate the ESR that occurred in the driving syringe, it was necessary to quantify the microscopic image intensity of the blood sample flowing in the blood sample channel. A specific ROI with 300 × 150 pixels was selected in the blood sample channel. The image intensity of the blood sample flowing in the blood sample channel was obtained by conducting digital image processing with MATLAB. An averaged image intensity (<*I_BC_*>) was obtained by averaging variations of *I_BC_* distributed over the ROI.

### 2.4. Discrete Fluidic Circuit for Representing Viscoelasticity of Blood Sample

Blood samples were assumed to be Newtonian fluids. To evaluate the viscoelasticity of the blood sample, a simple mathematical model was constructed with discrete fluidic circuit elements. As shown in Figure 1B-c, two fluids flowing in the coflowing channel (i.e., blood sample stream, reference fluid stream) are represented with individual discrete fluidic circuit elements. The fluid circuit model is composed of a flow-rate element (*Q_B_*, *Q_R_*), resistance element (*R_B_* and *R_R_*), and compliance element (*C_B_*). *Q_B_* and *Q_R_* are the flow rates of the blood sample and reference fluid, respectively. Ground represented zero value of pressure (*P* = 0). 

The blood stream for representing the viscoelasticity of the blood sample was modeled as a resistance element (*R_B_*) and compliance element (*C_B_*) combined in parallel. The *C_B_* was included to account for the compliance effect of the RBCs, the microfluidic channel, and a connected tube. However, because the reference fluid flowed at a constant flow rate in the coflowing channel, the compliance effect of a microfluidic channel and tube filled with reference fluid was negligible. For this reason, the reference stream was simply modeled only as a single resistance element (*R_R_*). The coflowing channel was partially filled with the blood sample stream (*W_B_*) and reference fluid stream (*W* – *W_B_*). Because ground represented zero value of pressure, both streams had the same pressure drop (*P_B_* = *P_R_* = Δ*P* = *P*). Based on mass conservation for the blood sample stream and reference fluid stream in the coflowing channel, two equations were derived:

(1) $$Q_{B}=\frac{P}{R_{B}}+C_{B}\frac{dP}{dt}$$

for the blood sample stream, and

(2) $$Q_{R}=\frac{P}{R_{R}}$$

for the reference fluid stream. By inserting Equation (2) into Equation (1), a first-order ordinary differential equation was derived:

(3) $$\frac{Q_{B}}{Q_{R}}=\frac{R_{R}}{R_{B}}+C_{B}\frac{d}{dt}\left(R_{R}\right)$$

Because a rectangular-shaped channel (width = *w*, depth = *h*, and length = *l*) with a lower aspect ratio (*AR*) (i.e., *AR* = depth/width = 20/250) was filled with fluid (viscosity = *μ*), the resistance element was modeled approximately as [21]:

(4) $$R=\frac{12\;\mu \;L}{w\;h^{3}}$$

Based on the analytical expression of a rectangular channel, the corresponding resistance element for each stream was derived as

(5) $$R_{B}=\frac{12\mu_{B}l_{cc}}{W\alpha_{B}\;h^{3}}$$

for the blood sample stream and

(6) $$R_{R}=CF\times \frac{12\mu_{R}l_{cc}}{W(1-\alpha_{B})\;h^{3}}$$

for the reference fluid stream. In Equation (6), the *CF* was included to compensate for the boundary condition difference between the real physical model and approximate circuit model [17]. When inserting Equations (5) and (6) into Equation (4), the following equation was derived.

(7) $$\frac{Q_{B}}{Q_{R}}=C_{B}\frac{d}{dt}\left(CF\frac{12\mu_{R}l_{cc}}{W\left(1-\alpha_{B}\right)h^{3}}\right)+CF\left(\frac{\mu_{R}}{\mu_{B}}\right)\left(\frac{\alpha_{B}}{1-\alpha_{B}}\right)$$

The first part in the right side of Equation (7) was expressed again in a different form.

(8) $$C_{B}\frac{d}{dt}\left(CF\frac{12\mu_{R}l_{cc}}{W\left(1-\alpha_{B}\right)h^{3}}\right)=C_{B}\frac{d}{dt}\left(CF\times R_{WB}\times \frac{1}{\left(1-\alpha_{B}\right)}\times \frac{\mu_{R}}{\mu_{B}}\right)$$

In Equation (8), *R_WB_*, which assumed that the coflowing channel was filled with the blood sample, was given as $R_{WB}=\frac{12\mu_{B}l_{cc}}{Wh^{3}}$. According to a previous study, blood viscosity remained constant with respect to the interface [18]. Because *R_WB_* and $\frac{\mu_{R}}{\mu_{B}}$ were independent of time, Equation (8) became a simple expression of Equation (9).

(9) $$C_{B}\frac{d}{dt}\left(CF\frac{12\mu_{R}l_{cc}}{W\left(1-\alpha_{B}\right)h^{3}}\right)=C_{B}\times R_{WB}\times \left(\frac{\mu_{R}}{\mu_{B}}\right)\times \frac{d}{dt}\left(CF\times \frac{1}{\left(1-\alpha_{B}\right)}\right)$$

When Equation (9) was inserted into Equation (7), Equation (7) was then expressed as Equation (10).

(10) $$\left(\frac{Q_{B}}{Q_{R}}\right)\left(\frac{\mu_{B}}{\mu_{R}}\right)=C_{B}R_{WB}\frac{d}{dt}\left(CF\times \frac{1}{1-\alpha_{B}}\right)+CF\times \left(\frac{\alpha_{B}}{1-\alpha_{B}}\right)$$

In Equation (10), *CF* was varied depending on the interface (*α_B_*) (i.e., *CF* = *CF* (*α_B_*)). Here, the *CF* was obtained by conducting numerical simulation. Because Equation (10) had nonlinear terms, it was substantially difficult to find an analytical solution. For this reason, it was necessary to approximate the nonlinear Equation (10) as a simple linear equation. Equation (10) was modified as a simple form.

(11) $$\left(\frac{Q_{B}}{Q_{R}}\right)\left(\frac{\mu_{B}}{\mu_{R}}\right)=F_{1}C_{B}R_{WB}\frac{d}{dt}\left(\frac{1}{1-\alpha_{B}}\right)+F_{2}\left(\frac{\alpha_{B}}{1-\alpha_{B}}\right)$$

In Equation (11), *F_1_* and *F_2_* were obtained by obtaining the weighted average of *CF* (i.e., *CF* × (1 − *α_B_*)^−1^ or *CF* × *α_B_* × (1 − *α_B_*)^−1^) within a specific value of the interface. As the interface was relocated periodically within a specific range (0.1 < *α_B_* < 0.9), two approximate factors (*F_1_* and *F_2_*) with constant values were calculated from Equations (12) and (13).

(12) $$\sum_{i=1}^{i=n}CF\left(\alpha_{B}\left[i\right]\right)\times \frac{1}{1-\alpha_{B}\left(i\right)}=F_{1}\sum_{i=1}^{i=n}\frac{1}{1-\alpha_{B}\left(i\right)}$$

and

(13) $$\sum_{i=1}^{i=n}CF\left(\alpha_{B}\left[i\right]\right)\times \frac{\alpha_{B}\left(i\right)}{1-\alpha_{B}\left(i\right)}=F_{2}\sum_{i=1}^{i=n}\frac{\alpha_{B}\left(i\right)}{1-\alpha_{B}\left(i\right)}$$

By dividing Equation (11) with *F_2_*, Equation (11) was changed to a simple linear differential equation.

(14) $$\lambda_{B}\frac{d}{dt}\left(\beta_{B}\right)+\beta_{B}=1+\left(\frac{1}{F_{2}}\right)\left(\frac{Q_{B}}{Q_{R}}\right)\left(\frac{\mu_{B}}{\mu_{R}}\right)$$

In Equation (14), $\beta_{B}$ and time constant ($\tau_{B}$) were derived as $\beta_{B}={\left(1-\alpha_{B}\right)}^{-1}$ and $\lambda_{B}=C_{B}R_{WB}\left(\frac{F_{1}}{F_{2}}\right)$, respectively. In this study, the flow rates of the blood sample and reference fluid were controlled as $Q_{B}\left(t\right)=Q_{0}+Q_{1}\sin\left(\omega t\right)$ and $Q_{R}\left(t\right)=Q_{0}$, respectively. Here, ω was given as $\omega =\frac{2\pi }{T}$. The particular solution of Equation (14) was then derived as

(15) $$\beta_{B}=\beta_{0}+\beta_{1}\sin\left(\omega t-\varphi \right)$$

In Equation (15), $\beta_{0}$ and $\beta_{1}$ were given as Equations (16) and (17).

(16) $$\beta_{0}=1+\frac{1}{F_{2}}\left(\frac{\mu_{B}}{\mu_{R}}\right)$$

and

(17) $$\beta_{1}=\frac{1}{F_{2}}\left(\frac{\mu_{B}}{\mu_{R}}\right)\left(\frac{Q_{1}}{Q_{0}}\right)\frac{1}{\sqrt{1+\omega^{2}\lambda_{B}{}^{2}}}$$

Additionally, time delay (φ) was given as $\varphi =\omega \lambda_{B}$. From Equation (16), the blood viscosity (*μ_B_*) was given as

(18) $$\mu_{B}=\mu_{R}F_{2}\left(\beta_{0}-1\right)$$

Additionally, from Equation (17), the time constant ($\lambda_{B}$) was derived as

(19) $$\lambda_{B}=\frac{T}{2\pi }\sqrt{{\left(\frac{1}{\beta_{1}F_{2}}\right)}^{2}{\left(\frac{Q_{1}}{Q_{0}}\right)}^{2}{\left(\frac{\mu_{B}}{\mu_{R}}\right)}^{2}-1}$$

According to $\lambda_{B}=R_{WB}C_{B}\left(\frac{F_{1}}{F_{2}}\right)$, the analytical expression of compliance ($C_{B}$) was derived as

(20) $$C_{B}=\left(\frac{F_{2}}{F_{1}}\right)\left(\frac{1}{R_{WB}}\right)\left(\frac{T}{2\pi }\right)\sqrt{{\left(\frac{1}{\beta_{1}F_{2}}\right)}^{2}{\left(\frac{Q_{1}}{Q_{0}}\right)}^{2}{\left(\frac{\mu_{B}}{\mu_{R}}\right)}^{2}-1}$$

In other words, if $\beta_{0}$ and $\beta_{1}$ could be obtained from periodic variations of the interface ($\alpha_{B}$) in the coflowing channel, $\mu_{B}$ and *C_B_* as blood viscoelasticity could be evaluated from Equations (18) and (20), respectively.

As shown in Figure 1C, as a preliminary study, a blood sample (*Hct* = 50%) was prepared by adding normal RBCs into 1x PBS. The blood sample was supplied into inlet (a) at a sinusoidal flow rate (*Q_B_* (t) = 1 + 0.5 sin (2π*t*/360) mL/h). Simultaneously, 1x PBS was supplied into inlet (b) at a constant flow rate of *Q_R_* (*t*) = 1 mL/h. Figure 1C-a showed temporal variations of <*U_BC_*> and *α_B_* over time. In addition, <*U_BC_*> and *α_B_* exhibited periodic variations over time. Figure 1C-b showed temporal variations of *β_B_* = (1 − *α_B_*)^−1^ over time. Figure 1C-c showed temporal variations of *β_B_* selected for a single period (*T* = 360 s). The expression of *β_B_* (*t*) was assumed to be *β_B_* (t) = *a_0_* + *a_1_*·cos (ω*t*) + *a_2_*·sin (ω*t*). Using the orthogonal property of the sinusoidal function (sin (ω*t*) and cos (ω*t*)), three unknown constants (*a_0_*, *a_1_*, and *a_2_*) were obtained from Equations (21)–(23).

(21) $$a_{0}=\frac{1}{T}\int_{t=0}^{t=T}\beta_{B}\left(t\right)dt$$

(22) $$a_{1}=\frac{2}{T}\int_{t=0}^{t=T}\beta_{B}\left(t\right)\cos\left(\omega t\right)dt$$

and

(23) $$a_{2}=\frac{2}{T}\int_{t=0}^{t=T}\beta_{B}\left(t\right)\sin\left(\omega t\right)dt$$

According to Equations (21)–(23), three unknown constants (*a_0_*, *a_1_*, and *a_2_*) were obtained as $a_{0}=2.61$, $a_{1}=-0.758$, and $a_{2}=-0.017$. In Equation (15), $\beta_{0}$ and $\beta_{1}$ were obtained as $\beta_{0}=a_{0}=2.61$ and $\beta_{1}=\sqrt{a_{1}{}^{2}+a_{2}{}^{2}}=0.785$, respectively. Using Equations (18)–(20), blood viscosity, time constant, and blood compliance were estimated to be $\mu_{B}=1.785\;cP$, $\lambda_{B}=20.491\;s$, and *C_B_* = 208.492 $\frac{\mu m^{3}}{mPa}$, respectively. 

## 3. Results and Discussion

### 3.1. Correction Factor (CF) and Approximate Factors (F_1_ and F_2_) via Numerical Simulation

**Figure 2:**
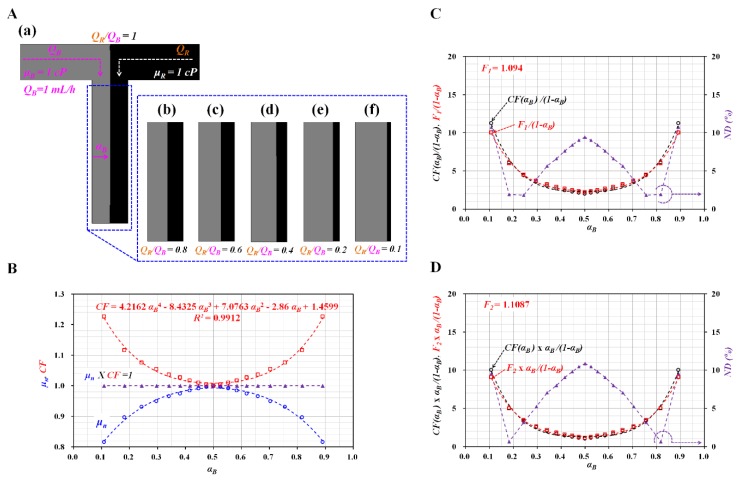
Estimation of correction factor (*CF*) and two approximate factors (*F_1_*, *F_2_*) with numerical simulation. (**A**) Variations of interface (*α_B_*) through numerical simulation with respect to flow-rate ratio (*Q_R_/Q_B_*): (**a**) *Q_R_/Q_B_* = 1, (**b**) *Q_R_/Q_B_* = 0.8, (**c**) *Q_R_/Q_B_* = 0.6, (**d**) *Q_R_/Q_B_* = 0.4, (**e**) *Q_R_/Q_B_* = 0.2, and (**f**) *Q_R_/Q_B_* = 0.1. For convenience, the flow rate of the blood sample was assumed to be *Q_B_* = 1 mL/h. The viscosities of both fluids were given as *μ_B_* = 1 cP and *μ_R_* = 1 cP, respectively. (**B**) Variations of estimated normalized viscosity (*μ_n_*) of blood sample and *CF* with respect to interface (0.1 < *α_B_* < 0.9). The *CF* was obtained as *CF* = 4.2162 *α_B_^4^* – 8.4325 *α_B_^3^ + 7.0763 α_B_^2^* − 2.86 *α_B_^2^* + 1.4599 (*R^2^* = 0.9912). (**C**) Variations of *CF (α_B_)/(1 − α_B_)*, *F_1_/(1 − α_B_)*, and normalized difference (*ND*) over *α_B_*. (**D**) Variations of *CF (α_B_)* × *α_B_/(1 −α_B_)*, *F_2_* × *α_B_/(1 − α_B_)*, and *ND* over *α_B_*.

To find two approximate factors (*F_1_*, *F_2_*) in Equation (11), it was necessary to obtain the *CF* by conducting numerical simulation with CFD software (CFD-ACE+, ESI Group, Paris, France). For convenience, the flow rate of the blood sample was assumed to be *Q_B_* = 1 mL/h. The viscosities of both fluids (blood sample, reference fluid) were assumed as *μ_B_* = 1 cP and *μ_R_* = 1 cP, respectively. Figure 2A showed variations of the interface (*α_B_*) through numerical simulation with respect to the flow-rate ratio (*Q_R_/Q_B_*) ((a) *Q_R_/Q_B_* = 1, (b) *Q_R_/Q_B_* = 0.8, (c) *Q_R_/Q_B_* = 0.6, (d) *Q_R_/Q_B_* = 0.4, (e) *Q_R_/Q_B_* = 0.2, and (f) *Q_R_/Q_B_* = 0.1). The interfaces (*α_B_*) for the corresponding flow-rate ratio (*Q_R_/Q_B_*) were obtained as (a) *α_B_* = 0.5 for *Q_R_/Q_B_* = 1, (b) *α_B_* = 0.553 for *Q_R_/Q_B_* = 0.8, (c) *α_B_* = 0.619 for *Q_R_/Q_B_* = 0.6, (d) *α_B_* = 0.704 for *Q_R_/Q_B_* = 0.4, (e) *α_B_* = 0.818 for *Q_R_/Q_B_* = 0.2, and (f) *α_B_* = 0.891 for *Q_R_/Q_B_* = 0.1. 

According to the coflowing-streams method [22,23], the viscosity ratio of the blood sample to the reference fluid (*μ_B_*/*μ_R_*) was obtained as $\frac{\mu_{B}}{\mu_{R}}=\frac{\alpha_{B}}{1-\alpha_{B}}$ by quantifying the interface (*α_B_*) in coflowing streams at the same flow-rate condition (*Q_B_* = *Q_R_*). The normalized viscosity of the blood sample was obtained by dividing the estimated viscosity (*μ_est_* = *μ_R_*·*α_B_*/(1 − *α_B_*)) by the given viscosity (*μ_given_* = 1 cP) (*μ_n_* = *μ_est_*/*μ_given_*). As shown in Figure 2B, variations of *μ_n_* were obtained by varying the interface (0.1 < *α_B_* < 0.9). When the interface was located at the center line (*α_B_* = 0.5), *μ_n_* was given as *μ_n_* = 1. In other words, the blood viscosity could be measured accurately when the interface was located at the center line [24]. However, when *α_B_* was relocated from the center line to both walls, *μ_n_* tended to decrease by approximately 0.8. Because the viscosity of the blood sample was given as *μ_B_* = 1 cP, the coflowing-streams method exhibited a large measurement error of approximately 20% when compared with the given viscosity of the blood sample. The reason could be explained by the boundary condition difference between the real physical model and simple mathematical model. Instead of a real and complex model, to construct a simple model of coflowing streams, the coflowing-streams method assumed that the interface of two streams was a virtual wall. In other words, it assumed that the interface was a virtual-wall boundary. Because the *CF* varied by channel dimensions (width and depth), the *CF* was calculated by referring to the general procedure discussed in previous studies [17,19]. The *CF* was then estimated by the reciprocating *μ_n_* obtained at a specific interface (*CF·(α_n_)* = *μ_n_^−1^* for *α_n_*). As shown in Figure 2B, the *CF* was obtained as *CF* = 1 at the center line (*α_B_* = 0.5). However, the *CF* tended to increase gradually when the interface moved to both walls. According to regression analysis, the *CF* was obtained as *CF* = 4.2162 *α_B_^4^* – 8.4325 *α_B_^3^* + 7.0763 *α_B_^2^* − 2.86 *α_B_* + 1.4599 (*R^2^* = 0.9912). By inserting the correction factor into the coflowing-streams method, the viscosity of the blood sample was estimated with $\mu_{B}=\mu_{R}CF\left(\alpha_{B}\right)\frac{\alpha_{B}}{1-\alpha_{B}}$. The coflowing method with correction factor could be used to measure the viscosity of blood with a specific hematocrit. Based on Equation (12), the approximate factor (*F_1_*) was obtained as *F_1_* = 1.094. As shown in Figure 2C, variations of $CF\left(\alpha_{B}\right)\frac{1}{1-\alpha_{B}}$ and $F_{1}\frac{1}{1-\alpha_{B}}$ were obtained with respect to α_B_. The normalized difference (*ND*) between both terms exhibited its maximum value at the center and both walls. The normalized difference was less than 10%. Additionally, according to Equation (13), the approximate factor (*F_2_*) was obtained as *F_2_* = 1.1087. As shown in Figure 2D, variations of $CF\left(\alpha_{B}\right)\frac{\alpha_{B}}{1-\alpha_{B}}$ and $F_{2}\frac{\alpha_{B}}{1-\alpha_{B}}$ were obtained with respect to α_B_. The maximum value of normalized difference was estimated to be approximately 11%. The simulation study showed that the two approximate factors (*F_1_* =1.094, *F_2_* =1.1087) could give consistent results when compared with the original expression. Using *F_1_* and *F_2_*, the nonlinear Equation (10) was converted into the simple linear Equation (11) for consistency. 

### 3.2. Effect of Period (T) on Viscoelasticity of Blood Sample

To verify the contribution of the period (*T*) to the viscoelasticity of the blood sample (Equations [18] and [20]), the viscosity and compliance were evaluated by varying the period (*T* = 120, 240, 360, and 480 s). The blood sample (*Hct* = 50%) was prepared by adding normal RBCs into 1x PBS. *Q_0_* and *Q_1_* of the two syringe pumps were controlled at *Q_0_* = 1 and *Q_1_* = 0.5 mL/h, respectively. For a rectangular channel with a lower aspect ratio, the shear rate ($\dot{\gamma }$) was derived as $\dot{\gamma }=\frac{6Q}{wh^{2}}$ [19]. Based on the shear rate formula, the shear rates of the corresponding flow rate were estimated as $\dot{\gamma }=8333{\;s}^{-1}$ for *Q_B_* = 0.5 mL/h and $\dot{\gamma }=25,000{\;s}^{-1}$ for *Q_B_* = 1.5 mL/h. Because the shear rate ($\dot{\gamma }$) was much greater than 1000 s^-1^, it was reasonable that the blood sample behaves as a Newtonian fluid. In other words, the blood viscosity (*μ_B_*) remained constant within the specific flow rates of the blood sample. 

**Figure 3:**
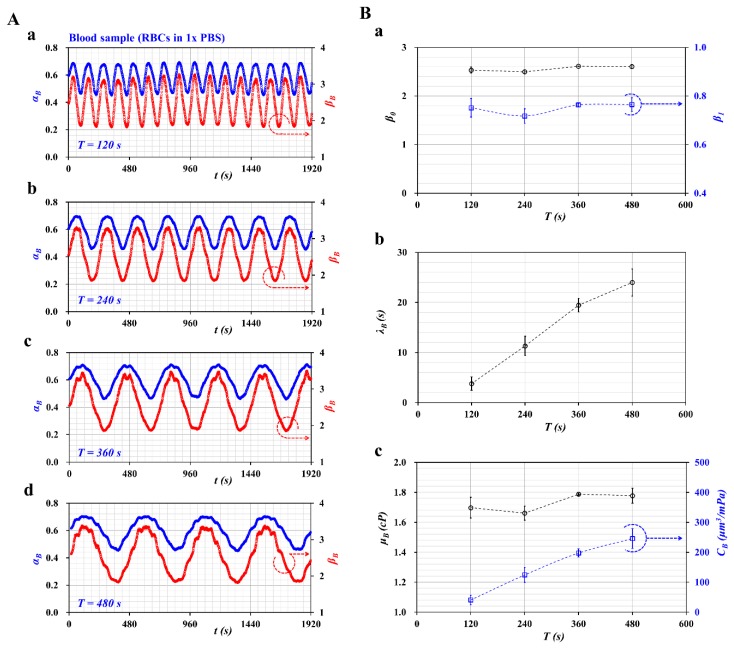
Evaluation of the effect of period (*T*) on viscoelasticity (blood viscosity and compliance). (**A**) Temporal variations of *α_B_* and *β_B_* with respect to period (*T*) ((**a**) *T* = 120 s, (**b**) *T* = 240 s, (**c**) *T* = 360 s, and (**d**) *T* = 480 s). (**B**) Quantification of viscosity (*μ_B_*) and compliance (*C_B_*) with respect to period. (**a**) variations of *β_0_* and *β_1_* with respect to *T*, (**b**) variations of *λ_B_* with respect to *T*, and (**c**) variations of *μ_B_* and *C_B_* with respect to *T*.

As shown in Figure 3A, temporal variations of *α_B_* and *β_B_* = (1 − *α_B_*)^−1^ were obtained with respect to the period ((a) *T* = 120 s, (b) *T* = 240 s, (c) *T* = 360 s, and (d) *T* = 480 s). Based on Equations (21)–(23), *β_0_* and *β_1_* were obtained at an interval of the corresponding period. Figure 3B-a showed variations of *β_0_* and *β_1_* with respect to *T*, where *β_0_* and *β_1_* fluctuated at a shorter period (*T* = 120, 360 s). However, they remained stable at a longer period (*T* = 360, 480 s). Figure 3B-b showed variations of *λ_B_* with respect to *T*. The *λ_B_* tended to increase linearly for up to *T* = 360 s. However, the slope of *λ_B_* tended to decrease between *T*= 360 and *T* = 480 s. According to Equation (19), the *λ_B_* was linearly proportional to the period (i.e., *λ_B_* ~ *T*). The experimental results showed appropriately consistent variations of *λ_B_* with respect to *T*. Using Equations (18) and (20), the blood viscosity (*μ_B_*) and compliance (*C_B_*) were obtained with respect to *T*. As shown in Figure 3B-c, *μ_B_* did not exhibit a linear dependency of *T*. It fluctuated at a shorter period. However, it remained constant at a longer period (*T* = 360, 480 s). The results agreed with Equation (18), which did not relate to the period. It was necessary, however, to set a longer period for consistently measuring the viscosity of the blood sample. Compliance (*C_B_*) tended to increase linearly for up to *T* = 360 s. The slope of *C_B_* tended to decrease between *T* = 360 and *T* = 480 s. According to the mathematical relation, *C_B_* was linearly proportional to *λ_B_*. The experimental results indicated that blood viscosity was independent of period. However, compliance varied linearly depending on the period.

From the results, for consistent measurement of viscoelasticity (blood viscosity and compliance), the period of the syringe pump was set to a longer period of *T* = 360 s throughout all experiments for convenience.

### 3.3. Quantification of the Effect of Hematocrit on Blood Viscoelasticity

According to a previous study, hematocrit caused an increase in blood viscosity and elasticity [16]. In addition, a blood sample with a low hematocrit (*Hct* = 30%) exhibited a continuous ESR occurring in a driving syringe [25]. According to the previous study, to increase the ESR significantly, a blood sample was prepared by adding normal RBCs into various concentrations of dextran solution (*C_dex_* = 2, 5, 8, and 10 mg/mL). Because the hematocrit of the blood sample flowing in the microfluidic channel varied continuously over time, RBC aggregation or blood viscosity tended to vary continuously [19]. In this study, under blood perfusion with a sinusoidal flow-rate pattern, the contribution of hematocrit to blood viscoelasticity and ESR was quantified by varying the hematocrit. To induce the ESR in a driving syringe, plasma was used as the diluent. In other words, blood samples (*Hct* = 30%, 40%, 50%, and 60%) were prepared by adding normal RBCs into the plasma.

**Figure 4:**
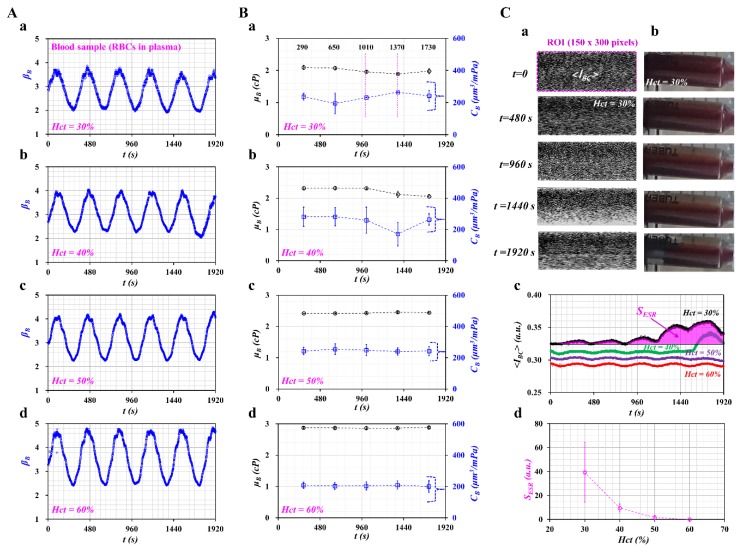
Contribution of hematocrit (*Hct*) to viscoelasticity and ESR. Blood samples (*Hct* = 30%, 40%, 50%, and 60%) were prepared by adding normal RBCs into plasma. (**A**) Temporal variations of *β_B_* with respect to hematocrit ((**a**) *Hct* = 30%, (**b**) *Hct* = 40%, (**c**) *Hct* = 50%, and (**d**) *Hct* = 60%). (**B**) Temporal variations of *μ_B_* and *C_B_* with respect to hematocrit ((**a**) *Hct* = 30%, (**b**) *Hct* = 40%, (**c**) *Hct* = 50%, and (**d**) *Hct* = 60%). (**C**) Evaluation of ESR occurring in driving syringe. (**a**) Microscopic images of blood sample (*Hct* = 30%) flowing in blood channels over time (*t* = 0, 480, 960, 1440, and 1920 s). (**b**) Side view of a driving syringe over time (*t* = 0, 480, 960, 1440, and 1920 s). (**c**) Temporal variations of *<I_BC_>* with respect to *Hct.* (**d**) Variations of *S_ESR_* with respect to *Hct*.

Figure 4A showed temporal variations of *β_B_* with respect to hematocrit ((a) *Hct* = 30%, (b) *Hct* = 40%, (c) *Hct* = 50%, and (d) *Hct* = 60%). Using temporal variations of *β_B_*, blood viscosity and compliance were obtained at an interval of a specific period. As shown in Figure 4B, temporal variations of *μ_B_* and *C_B_* were obtained with respect to hematocrit ((a) *Hct* = 30%, (b) *Hct* = 40%, (c) *Hct* = 50%, and (d) *Hct* = 60%). Both parameters were obtained as mean ± standard deviation at a specific time (*t* = 290, 650, 1010, 1370, and 1730 s).

For the blood sample with *Hct* = 30%, compliance (*C_B_*) fluctuated greatly over time. It tended to decrease after *t* = 290 s, but it tended to increase between *t* = 650 and *t* = 1370 s. After *t* = 1370 s, it remained constant over time. Blood viscosity tended to decrease after *t* = 650 s. In other words, the ESR of RBCs in a driving syringe accelerated over time. In other words, after a certain time, the hematocrit of the blood sample tended to decrease. Thus, *μ_B_* tended to decrease, and *C_B_* tended to increase with an elapse of time. For the blood sample with *Hct* = 40%, blood viscosity tended to decrease after *t* = 1010 s. Compliance remained constant for up to *t* = 1010 s. After that, it fluctuated over time. When the hematocrit increased from *Hct* = 30% to *Hct* = 40%, the time when *C_B_* had the minimum value tended to increase substantially. However, for the blood samples with high hematocrit (*Hct* = 50%, 60%), blood viscosity and compliance remained constant over time. The blood viscosity tended to increase at a higher hematocrit. The compliance tended to decrease at a higher hematocrit. According to a previous study, blood elasticity tended to increase with respect to hematocrit [16]. When compared with the previous result, compliance tended to decrease with respect to the hematocrit. The experimental results can be considered reasonable, because compliance had the reciprocal of elasticity (i.e., compliance ~ 1/elasticity). 

To quantify the ESR that occurred in a driving syringe, the microscopic image intensities of the blood sample were obtained over time. A driving syringe was installed horizontally. Because of the ESR in the driving syringe, the hematocrit of the blood sample flowing in a microfluidic channel tended to decrease over time. Here, variations of hematocrit were used by monitoring the image intensity of the blood sample [26]. Figure 4C-a showed microscopic images of the blood sample (*Hct* = 30%) flowing in the blood sample channel over time (*t* = 0, 480, 960, 1440, and 1920 s). After *t* = 960 s, the number of RBCs tended to decrease significantly. The image intensity tended to increase. At *t* = 1920 s, the image intensity tended to decrease as RBCs tended to increase significantly. The contrast of each image was enhanced by conducting image processing with the software Image-J (NIH, Maryland, USA). A specific ROI (300 x 150 pixels) in the blood sample channel was selected to quantify the image intensity (<*I_BC_*>). To visualize the ESR in a driving syringe, a side view of a syringe filled with a blood sample (*Hct* = 30%) was captured sequentially with a smartphone camera (Galaxy A5, Samsung, Korea). As lower level of hematocrit exhibited higher value of *<I_BC_>*, the results of blood sample (*Hct* = 30%) was selected and summarized at a specific time. Figure 4C-b showed snapshots of a driving syringe over time (*t*) (*t* = 0, 480, 960, 1440, and 1920 s). Before *t* = 480 s, there was no indication of the ESR occurring in a driving syringe, because the RBCs were distributed uniformly. After *t* = 960 s, because of the continuous ESR, the blood sample inside the syringe was separated into two regions: an RBC-rich region (i.e., lower layer) and an RBC-free region (i.e., upper layer). As shown in Figure 4C-a, the ESR in the driving syringe caused a reduced number of RBCs in the microfluidic channel. To quantify the decrease in hematocrit resulting from ESR in the driving syringe, the image intensity of the blood sample (<*I_BC_*>) was obtained over time. Figure 4C-c showed temporal variations of *<I_BC_>* with respect to *Hct* = 30%, 40%, 50%, and 60%. For a blood sample with *Hct* = 30%, <*I_BC_*> tended to increase after *t* = 1190 s. For a blood sample with *Hct* = 40%, image intensity tended to increase after *t* = 1570 s. For blood samples with a high hematocrit (*Hct* = 50%, 60%), the image intensity remained constant over time. This result showed that a lower hematocrit contributed to varying image intensity (or numbers of RBCs) of blood samples flowing in a microfluidic channel. According to a specific parameter (*S_ESR_*) suggested in a previous study [20], variations of ESR were quantified with respect to *Hct*. The *S_ESR_* was obtained as $S_{ESR}=\int_{t=0}^{t=1920}\left(\langle I_{BC}\rangle -I_{min}\right)dt$. Here, *I_min_* represents the minimum value of <*I_BC_*> within a specific duration, *t* = 1920 s. Figure 4C-d showed variations of *S_ESR_* with respect to *Hct*. The *S_ESR_* tended to decrease substantially with respect to *Hct*. The blood sample with *Hct* = 30% showed the maximum value of *S_ESR_*. This result indicated that *S_ESR_* varied significantly depending on the hematocrit.

From the result, one can conclude that the viscoelasticity of the blood sample suggested by the present method can be varied with the hematocrit. In addition, it can be employed to quantify variations of the ESR occurring in the driving syringe by monitoring temporal variations of viscoelasticity.

### 3.4. Quantification of the Contribution of Hardened RBCs to Blood Viscoelasticity

Finally, the method was employed to quantify the contribution of hardened RBCs to the viscoelasticity of blood samples. According to previous studies [27,28], normal RBCs were hardened chemically with GA solution. The degree in rigidity increased gradually by varying concentrations of the GA solution. Normal RBCs were hardened chemically with three different concentrations of GA solution (*C_GA_*) (*C_GA_* = 4, 8, and 12 μL/mL). The hardened blood sample (*Hct* = 50%) was then prepared by adding hardened RBCs into plasma.

**Figure 5:**
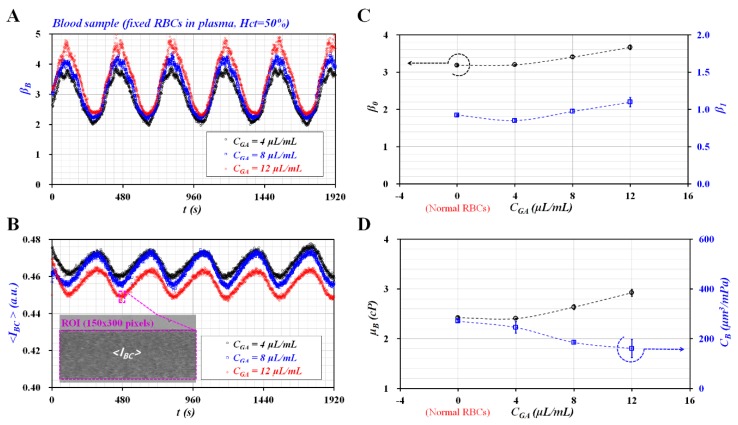
Contribution of fixed blood sample to viscoelasticity of blood sample. Fixed RBCs were prepared chemically with GA solution (*C_GA_*) (*C_GA_* = 4, 8, and 12 µL/mL). A fixed blood sample (*Hct* = 50%) was then prepared by adding fixed RBCs into plasma: (**A**) temporal variations of *β_B_* with respect to *C_GA_*, (**B**) temporal variations of <*I_BC_*> with respect to *C_GA_*, (**C**) variations of *β_0_* and *β_1_* with respect to *C_GA_*, and (**D**) variations of *μ_B_* and *C_B_* with respect to *C_GA_*.

Figure 5A showed temporal variations of *β_B_* with respect to *C_GA_*. As the concentration of GA solution increased, *β_B_* tended to increase gradually. In addition, the *β_B_* exhibited steady and periodic variations over time. Figure 5B showed temporal variations of <*I_BC_*> with respect to *C_GA_*. The inset showed a microscopic image of the fixed blood sample (i.e., fixed RBCs with *C_GA_* = 12 μL/mL) captured at *t* = 480 s. The <*I_BC_*> tended to decrease slightly at a higher concentration of GA solution. However, it exhibited steady and periodic variations over time. The result indicated that the fixed blood sample with *Hct* = 50% did not induce the ESR in the driving syringe as shown in Figure 4. 

Figure 5C showed variations of *β_0_* and *β_1_* with respect to *C_GA_*. Both parameters (*β_0_* and *β_1_*) tended to increase substantially at a higher concentration of GA solution. Because the GA solution was employed to increase the rigidity of RBCs, both parameters presented distinctive variations of hardness. Based on Equations (18)–(20), variations of *μ_B_* and *C_B_* were obtained at an interval of *T* = 360 s. As *β_B_* and <*I_BC_*> showed stable variations over time, *μ_B_* and *C_B_* remained constant over the specific duration of the test as shown in Figure 5A,B. Thus, *μ_B_* and *C_B_* were represented as average ± standard deviation (*n* = 5). Figure 5D showed variations of *μ_B_* and *C_B_* with respect to *C_GA_*. According to previous studies [17,18], blood viscosity and elasticity tended to increase at a higher concentration of GA solution. When compared with the previous results, blood viscosity exhibited consistent variations with respect to the concentration of GA solution. Because compliance was defined as the reciprocal of elasticity, the compliance also showed consistent variations with respect to the concentration of GA solution.

The results lead to the conclusion that the present method could be employed to monitor variations of the viscoelasticity of blood samples while the syringe pump was set to a pulsatile flow-rate pattern. 

### 3.5. Quantificative Comparison of Viscoelasticty Obtained with Preesent Method and Conventional Viscometer

**Figure 6:**
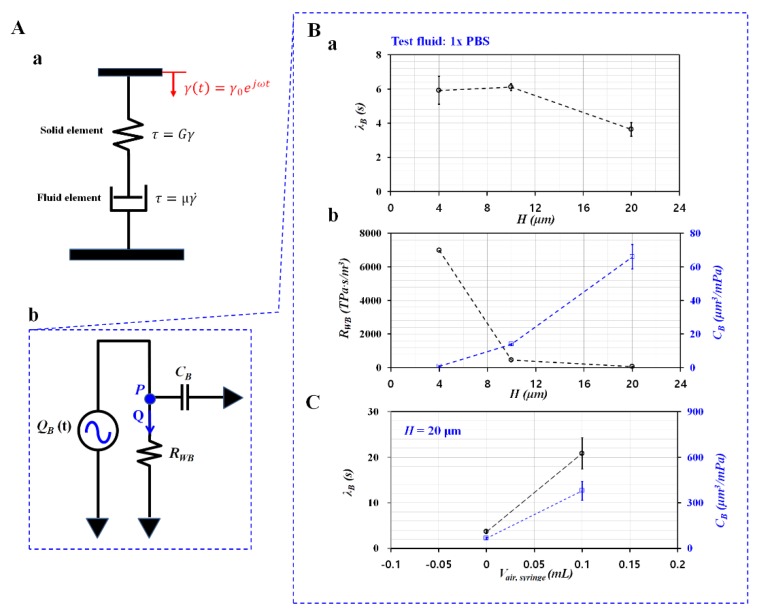
Mathematical representation and quantification of contributions of channel depth and air cavity to compliance. (**A**) Mathematical representation of conventional viscometer and microfluidic system. (**a**) Mathematical representation of conventional viscometer. (**b**) Mathematical representation of microfluidic system. (**B**) Evaluation of channel depth (*H*) on compliance. Here, 1x PBS as test fluid was infused into a microfluidic system. (**a**) Variations of *λ_B_* with respect to *H*. (**b**) Variations of *R_WB_* and *C_B_* with respect to *H*. (**C**) Evaluation of air cavity on compliance.

Using a conventional viscometer, viscoelasticity of cells was modeled with the linear Maxwell model. As shown in Figure 6A-a, the viscoelasticity of each blood sample was modeled as a solid element and a fluid element connected in series. The corresponding constitutive expression of each element was given as τ=Gγ (solid element) and $\tau =\mu \dot{\gamma }$ (fluid element), respectively. Here, *G* and *μ* represented elasticity and viscosity, respectively. *τ* and *γ* denoted shear stress and shear strain. External shear strain was excited periodically as $\gamma \left(t\right)=\gamma_{0}e^{j\omega t}$. The governing equation was then derived as $\dot{\tau }+\frac{1}{\lambda_{cv}}\tau =G\dot{\gamma }$. Here, the time constant of viscometry (*l_cv_*) was expressed as *l_cv_* = μ/G. The viscous effect of the viscometer was considered as negligible since the viscometer did not have an influence on relaxation time of the cells. The viscometer had operated at a wider range of frequency from ω = 0.3 rad/s to ω = 700 rad/s [29]. The previous study indicated that the time constant of whole blood was obtained as *λ_cv_* = 1.5–13.4 ms for shear flow [29], and *λ_cv_* = 114–259 ms for extensional flow [30]. 

On the other hand, under periodic blood flow in the microfluidic system, Figure 6A-b showed the simple fluidic circuit model of the microfluidic system. Based on electric circuit analysis, the governing equation of fluidic system was derived as $\lambda_{B}\dot{Q}+Q=Q_{B}\left(t\right)$. Here, *Q* represented the flow rate which passed through resistance element. Time constant (*λ_B_*) was derived as *λ_B_* = *R_WB_*∙*C_B_*. *R_WB_* and *C_B_* represented fluidic resistance of the coflowing channel filled with only blood sample and compliance, respectively. As shown in Figure 6A-b, a microfluidic system behaved as an *R-C* low pass filer. To effectively infuse alternating components in the periodic flow rate into a microfluidic system, the period of the excitation flow rate should be much longer than the time constant of the microfluidic system (i.e., *T* > *λ_B_*). In addition, the syringe pump used in this study did not infuse blood samples during short periods. From the experimental results as shown in Figure 3B, period (*T*) of the sinusoidal flow rate was fixed as *T* = 360 s.

The *λ_B_* was determined by *R_WB_* and *C_B_*. Flexible tubing and the PDMS channels tended to vary the time constant substantially [11]. A microfluidic channel with different channel depths (*H*) (*H* = 4, 10, and 20 μm) was prepared to change *R_WB_*. Here, 1x PBS was infused into a microfluidic channel to reduce or remove the viscoelastic effects. As shown in Figure 6B-a, the corresponding time constant for each channel depth was obtained as *λ_B_* = 5.92 ± 0.81 s (*H* = 4 μm), *λ_B_* = 6.11 ± 0.20 s (*H* = 10 μm), and *l_B_* = 3.64 ± 0.40 s (*H* = 20 μm). In addition, Figure 6B-b showed variations of *R_WB_* and *C_B_* with respect to *H*. A lower channel depth contributed to increasing *R_WB_*, and decreasing *C_B_*. The result indicated that fluidic resistance (*R_WB_*) had a strong influence on *C_B_*. To find out the contribution of compliance element (*C_B_*), the compliance of the microfluidic system increased intentionally by securing the air cavity inside the driving syringe. As shown in Figure 6C, variations of *λ_B_* and *C_B_* were obtained with respect to *V_air_* = 0 and 0.1 mL. When the air cavity of 0.1 mL existed inside the driving syringe, *λ_B_* and *C_B_* increased considerably as *λ_B_* = 20.84 ± 3.42 s and *C_B_* = 378.59 ± 62.11 s. From the result, the air cavity tended to increase *λ_B_* and *C_B_* substantially. When compared with viscometry data, the time constant (*λ_B_*) increased about O (10^2^) significantly because of the compliance effect of the microfluidic system (i.e., flexible tubing, PDMS channels, and the air cavities existing in the driving syringe). In addition, minimum threshold of *C_B_* (i.e., detection limit) was estimated as 66.05 ± 7.30 μm^3^/mPa at a specific condition (i.e., *V_air_* = 0, *H* = 20 μm). According to a previous study [17], time constants obtained with two different systems (i.e., *λ_PM_*: microfluidic system, *λ_CPV_*: conventional viscometer) were obtained and compared with respect to *C_glycerin_* = 10%, 20%, 30% and 40%. As glycerin solution did not include viscoelasticity, both time constants remained unchanged with respect to different concentrations of glycerin solution. However, the microfluidic system had a longer time constant with O (10^0^). The ratio of time constant between *λ_CPV_* and *λ_PM_* was obtained as *λ_CPV_*/*λ_PM_* = O (10^2^). To quantitatively determine elasticity obtained with both methods, a scatter plot was employed by plotting *G_PM_* on the vertical axis and *G_CPV_* on the horizontal axis. According to linear regression analysis, regression coefficient (*R^2^*) had a higher value (*R^2^* = 0.9617). This result indicated that the elasticity obtained with microfluidic system exhibited consistent variations with respect to *C_glycrin_* when compared with elasticity obtained with conventional viscometer. Thus, the microfluidic system could be employed to measure viscoelasticity effectively. The slope of 0.0022 indicated that elasticity obtained with the microfluidic system was much less than that obtained with the conventional viscometer.

According to order analysis, *C_B_* had an order of O (10^−13^) from the analytical expression of time constant. Here, O represented order. According to experimental results as shown in Figure 5, the blood sample (normal RBCs suspended in plasma, *Hct* = 50%) had *λ_B_* = 36.084 ± 0.713 s, *μ_B_* = 2.422 ± 0.028 cP, and *R_WB_* = 135.148 ± 2.03 TPa∙s/m^3^. The compliance (*C_B_*) was then obtained as *C_B_* = 270.598 ± 4.63 μm^3^/mPa. The corresponding order of each parameter was calculated as (1) O (10^1^) for *λ_B_*, (2) O (10^0^) for *μ_B_*, and O (10^12^) for *R_WB_*. As the unit of *C_B_* was expressed as *μm^3^/mPa*, the order of *C_B_* was calculated as O (10^−18^)/O (10^−3^) = O (10^−15^). Thus, *C_B_* obtained for the blood sample had an order of O (10^−13^). In order words, both approaches (i.e., analytical expression, and experimental data) exhibited the same order of O (10^−13^). Thus, the present method could be used to monitor *C_B_* of blood samples sufficiently. 

**Figure 7:**
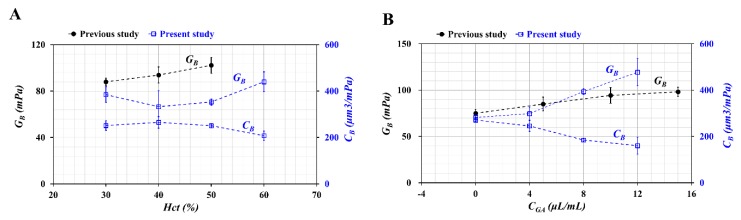
Quantitative compliance of *G_B_* and *C_B_* with respect to previous study [18] and present study. (**A**) Variation of *G_B_* and *C_B_* with respect to *Hct*. (**B**) Variations of *G_B_* and C_B_ with respect to *C_GA_*_._

To compare the relationship between *G_B_* and *C_B_*, it was assumed that *λ_cv_* of the conventional viscometer had the same *λ_B_* as the microfluidic system (i.e., $\lambda_{cv}=\lambda_{B}$). The time constant obtained by the microfluidic system was used to evaluate *G_B_* and *C_B_* simultaneously. The following relation was given as $\frac{\mu }{G}=R_{WB}\times C_{B}$. The analytical expression indicated that *G_B_* and *C_B_* had a reciprocal relationship (i.e., *G* ~ 1/*C_B_*). Using experimental results as shown in Figure 4, *G_B_* and *C_B_* were obtained with respect to *Hct* = 30%, 40%, 50%, and 60%. Additionally, by referring to the previous study [18], variations of *G_B_* were represented with respect to *Hct* = 30%, 40%, and 50%. Here, blood samples were prepared by adding normal RBCs into plasma. As shown in Figure 7A, at *Hct* > 40%, *G_B_* tended to increase with respect to *Hct*. Inversely, *C_B_* tended to decrease with respect to *Hct*. When compared with the previous study, the trend of *G_B_* increased similarly with respect to *Hct*. It can be inferred that different microfluidic systems contributed to differences of *G_B_* between both studies. Additionally, variations of *C_B_* and *G_B_* were summarized with respect to *C_GA_* as shown in Figure 7B. Here, in the previous study [4], fixed blood samples were prepared by adding fixed RBCs into 1x PBS instead of plasma. *G_B_* of both studies tended to increase gradually with respect to *C_GA_*. From quantitative comparisons between the previous study and the present study, elasticity (*G_B_*) and compliance (*C_G_*) had a reciprocal relationship. Additionally, they varied significantly when the rigidity of RBCs increased substantially.

Viscoelasticity (*G*) was represented as *G* = *G_1_*+ j *G_2_*. Here, *G_1_* (storing modulus) and *G_2_* (loss modulus) were expressed as $G_{1}=G\left(\frac{\lambda_{B}{}^{2}\omega^{2}}{1+\lambda_{B}{}^{2}\omega^{2}}\right)$ and $G_{2}=G\left(\frac{\lambda_{B}\omega }{1+\lambda_{B}{}^{2}\omega^{2}}\right)$. Variations of *G_1_* and *G_2_* were represented with respect to radial frequency (ω). *G_1_* and *G_2_* tended to vary depending on $\lambda_{B}$∙ω. However, as $\lambda_{B}$∙ω showed a significant difference for both systems, it was apparent that both systems exhibited different variations of *G_1_* and *G_2_* with respect to ω. The microfluidic system and conventional viscometer showed different characteristics in terms of angular frequency (or period) and time constants. However, according to experimental results, the microfluidic system could be used effectively to evaluate the viscoelasticity of human blood when compared with conventional viscometers.

## 4. Conclusions

In this study, a viscoelasticity measurement method for human blood samples (normal blood sample, fixed blood sample) was suggested by quantifying the interface in coflowing streams when a blood sample was infused at a sinusoidal flow rate. Using a discrete fluidic circuit model, a first-order nonlinear differential equation for the interface (*α_B_*) in coflowing streams was derived. Two approximation factors (*F_1_*, *F_2_*) were applied to convert a nonlinear term into a linear term. The viscosity and compliance (as viscoelasticity) were derived analytically from the linear differential equation. From numerical simulation, two approximate factors were obtained: *F_1_* = 1.094 and *F_2_* = 1.1087. The normalized difference between the nonlinear term and linear term was less than 10%. The experimental results showed that compliance varied linearly by period (*T*= 120, 240, 360, and 480 s). However, the blood viscosity remained constant with respect to period. The hematocrit and diluent contributed to varying viscoelasticity. Finally, viscoelasticity varied substantially depending on the degree in rigidity of RBCs. From the experimental results, it was found that the present method had the ability to monitor variations of viscoelasticity while the syringe pump was set to a pulsatile flow-rate pattern. As a limitation, the microfluidic device used in this study had microfluidic channels that were rectangular shape. However, human or animal blood vessels are not rigid and possess circular shape. When compared with live blood vessels, the rectangular shape of microfluidic device might contribute to varying viscoelasticity of the microfluidic channels within the blood sample. Microfluidic channels with 90° turns can cause live mammalian cells to lyse and aggregate in corners, causing fluid flow disruptions. In our next design, we will evaluate the contributions of channel shape and dimensions to viscoelasticity characteristics of the blood samples. In the near future, the present method will be employed to obtain the viscoelasticity of a blood sample circulated under ex vivo and in vivo conditions. In other words, to measure biomechanical properties of blood circulating under an extracorporeal bypass loop or hemodialysis, the blood sample will be collected from the fluidic circuit at periodic intervals. Biomechanical properties are then evaluated with individual conventional viscometers. However, the repetitive collection tends to reduce blood volume in the circuit substantially. If the present method will be integrated into the future fluidic circuit, viscoelasticity of the blood sample will be obtained at various intervals of time, even without periodic collection of blood samples. Blood volume loss might be reduced considerably in our microfluidic device when compared with the conventional method. Finally, the data obtained by the novel microfluidic system could be employed effectively to monitor vascular diseases and human health.

## Supplementary Materials

The following are available online at https://www.mdpi.com/2072-666X/11/3/245/s1, Figure S1: Mask drawing of silicon mold.
