# Supplementary material for: Blood Viscoelasticity Measurement Using Interface Variations in Coflowing Streams under Pulsatile Blood Flows
Source: Micromachines (Basel). 2020 Feb 26;11(3):245. doi: 10.3390/mi11030245 (PMC7142492; doi:10.3390/mi11030245)
Supplement: Supplementary file 1 [file micromachines-11-00245-s001.pdf]

micromachines

Supplementary Materials

Blood Viscoelasticity Measurement Using Interface Variations in Coflowing Streams under Pulsatile Blood Flows

Yang Jun Kang

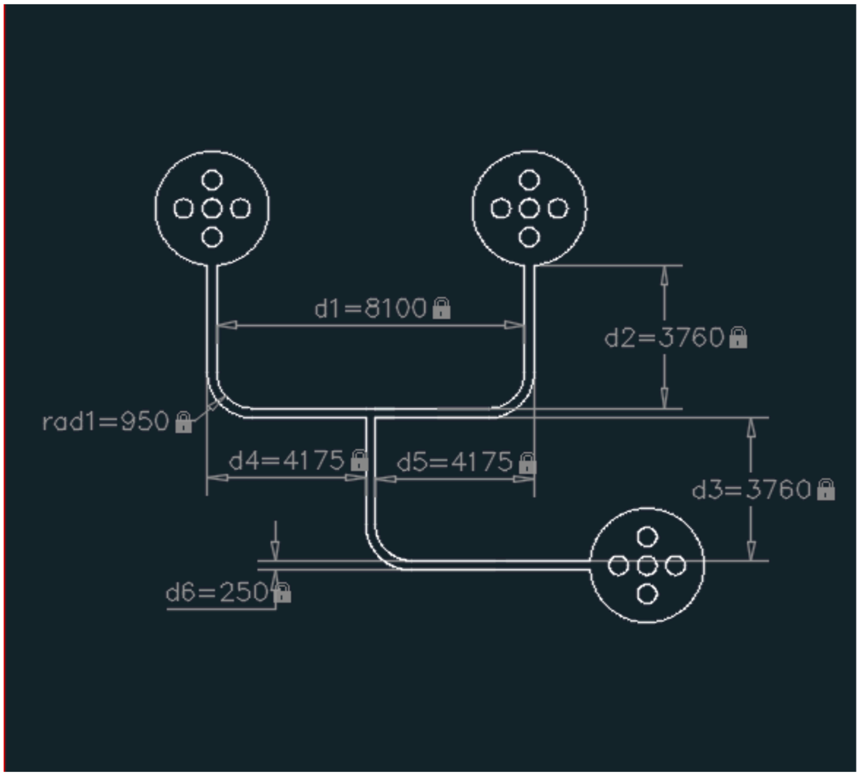

Figure S1. Mask drawing of silicon mold.

© 2020 by the authors. Submitted for possible open access publication under the terms and conditions of the Creative Commons Attribution (CC BY) license (http://creativecommons.org/licenses/by/4.0/).
